# Quality of life in Down syndrome in Brazil: a cross-sectional study

**DOI:** 10.1055/s-0043-1777006

**Published:** 2023-11-30

**Authors:** Beatriz Elizabeth Bagatin Veleda Bermudez, Gustavo Leite Franklin, Camila Maciel de Oliveira, Léo Coutinho, Ana Chrystina de Souza Crippa

**Affiliations:** 1 Universidade Federal do Paraná, Hospital das Clínicas, Ambulatório da Síndrome de Down, Curitiba PR, Brazil.; 2 Pontifícia Universidade Católica do Paraná, Faculdade de Medicina, Departamento de Medicina Interna, Curitiba PR, Brazil.; 3 Stanford University - Department of Science Behavior, Division of Sleep Medicine, Redwood City CA, United of States.; 4 Universidade Federal do Paraná, Programa de Pós-Graduação em Medicina Interna, Curitiba PR, Brazil.; 5 Universidade Federal do Paraná, Hospital das Clínicas, Unidade de Neuropediatria, Curitiba PR, Brazil.

**Keywords:** Down Syndrome, Intellectual Disability, Quality of Life, Comprehensive Health Care, Síndrome de Down, Deficiência Intelectual, Qualidade de Vida, Atenção Integral à Saúde

## Abstract

**Background** Down syndrome is the most commonly genetic cause of developmental delay and intellectual disability, affecting 1:700 live births. It is associated with heart disease and recurrent infections, among other complications that greatly impair the patient's quality of life.

**Objective** To evaluate the major factors associated with quality of life in a cohort of patients with Down syndrome.

**Methods** We assessed 1,187 patients with Down syndrome, older than 4 years old, with an adaptation of the Personal Outcomes Scale validated for Portuguese language, interviewing patients, parents, and caregivers.

**Results** A bad quality of life was reported in 56.4% of the sample. The main factors associated with better quality of life were female sex, first medical visit before 4 months old, higher parental education, a professionally active mother, and prenatal care. The main factors associated with worse quality of life were family history of alcohol abuse and psychiatric disorders and comorbidity with autism and epilepsy.

**Conclusion** Clinical comorbidities such as autism and epilepsy carry a heavy burden among patients with Down syndrome, while factors related to family support, such as employment status and educational background of the parents, enhance quality of life. The factors associated with quality of life among patients with Down syndrome should be adequately evaluated in medical consultation and targeted in public health policies.

## Resumo

**Antecedentes** A síndrome de Down é a mais comum causa identificável de atraso de desenvolvimento e deficiência intelectual, afetando 1 a cada 700 nascidos vivos. Está associada a cardiopatias, infecções recorrentes e outras complicações que impactam significativamente a qualidade de vida dos pacientes.

**Objetivo** Avaliar os principais fatores associados a qualidade de vida em uma coorte de pacientes com Síndrome de Down.

**Métodos** Avaliamos 1.187 pacientes com síndrome de Down com mais de 4 anos de idade utilizando uma adaptação da versão validada para o português da Escala Pessoal de Resultados, entrevistando pacientes, pais e cuidadores.

**Resultados** Uma má qualidade de vida foi encontrada em 56.4% da amostra. Os principais fatores associados à melhor qualidade de vida foram sexo feminino, primeira consulta médica antes dos 4 meses de idade, maior nível educacional dos pais, mãe profissionalmente ativa e atenção pré-natal. Os principais fatores associados à pior qualidade de vida foram o histórico familiar de abuso de álcool e distúrbios psiquiátricos, além de comorbidade com autismo e epilepsia.

**Conclusão** As comorbidades clínicas como autismo e epilepsia levam a um maior impacto entre os pacientes com síndrome de Down, enquanto fatores relacionados ao apoio familiar, como situação profissional e formação educacional dos pais, estão associados à melhor qualidade de vida. Os fatores associados à qualidade de vida de pacientes com síndrome de Down devem ser adequadamente avaliados em consulta médica e alvo de políticas públicas de saúde.

## INTRODUCTION

Down syndrome (DS) is the most commonly identified genetic cause of developmental delay and intellectual disability. It is characterized by trisomy of the chromosome 21 in 95% of the cases, with the remaining 5% being attributable to translocations and/or mosaicisms. 1 Patients with DS also present a wide variety of comorbidities, including congenital heart defects, recurrent infections, hearing impairment, thyroid abnormalities, overweight, and neuropsychiatric conditions such as autism and epilepsy, impacting their quality of life. 2

The World Health Organization (WHO) defines quality of life as an individuals' perception of their position in life in the context of the culture and value systems in which they live and in relation to their goals, expectations, standards, and concerns”. It is a wide concept that includes physical and psychological health, level of independence, and social relationships. 3 Although this is a well-studied subject among patients with DS from other cultures, a comprehensive report of the factors underlining quality of life among Brazilian patients with DS is still lacking. The present study assessed the quality of life and its related factors in a cohort of Brazilian patients with DS.

## METHODS

This work is a cross-sectional study that was conducted according to the Strengthening the Reporting of Observational Studies in Epidemiology (STROBE) guidelines for reporting observational studies. 4

### Eligibility criteria

This study was carried out in a public tertiary care center for patients with DS in Curitiba, state of Paraná, Brazil. Patients above 4 years old with DS, as well as their parents and/or caretakers, on regular follow-up at our service were invited to take part in this study. Attended patients were consecutively invited during the consultations. Patients younger than 4 years old, and patients/parents from whom informed consent could not be obtained were not included.

### Assessment of quality of life and associated factors

To evaluate quality of life in our sample, we used a version of the Personal Outcomes Scale (POS) adapted for Portuguese language. 5 This scale has been previously translated and validated into the Portuguese language by Simões et al. and is suitable to evaluate patients older than 4 years old. 6 The POS uses a 3-point Likert scale to grade the patient's quality of life, both self-reported and through direct observation, on three factors (independence, social participation, and wellbeing), divided over eight domains (personal development, self-determination, interpersonal relations, social inclusion, rights, and emotional, physical, and material wellbeing). The scores are summed to reach the quality-of-life self-report index and quality of life observation index, but there are no validated cutoff points as a standardized measure of quality of life in patients with DS, which forced us to adapt the scale, using the responses to each domain to tailor the endpoints of quality of life.

We established a good quality of life when patients presented good overall development, autonomy for activities of daily living (for example, bathing alone), and practical life activities (such as waiting to be picked up in front of the school or making a payment at a cash machine), as well as an adequate insertion in society, school, and/or their work environment. We defined a bad quality of life as the presence of significant developmental delay, high levels of family dependency, and no opportunities for inclusion or insufficient social skills. In addition, we sought information directly, or from the patient's files, concerning sex, age, age of first medical consult, breastfeeding history, neonatal screening, parental sociodemographic profile, enrollment in a special school, associated clinical comorbidities, physical activity, psychomotor development, family medical history, and results of an ancillary investigation, such as brainstem-evoked response audiometry (BERA).

### Statistical analysis

Numerical variables are expressed by means and standard deviations, while categorical variables are expressed in terms of proportions and percentages. Comparison between groups was established through Student *t* -test, Mann-Whitney test, Fisher exact test, and Pearson chi-square test, using the Statistica (StatSoft Inc., Tulsa, OK, USA) software. Missing data were handled by listwise deletion. The level of statistical significance was fixed at *p* < 0.005.

### Ethical considerations

This study was reviewed and approved by the Research Ethics Committee of Universidade Federal do Paraná. All patients and/or their tutors provided written informed consent to take part in this study.

## RESULTS

We gathered a sample of 1,187 patients, with a predominance of males (55.3% of the sample). Table 1 describes the age and sex distribution of the sample. Clinical and socioeconomic variables of interest to our sample, as well as their distribution in the groups with good or bad quality of life are summarized in Tables 2 , 3 , and 4 . The patients presented a predominantly bad quality of life, comprehending 56.4% of the sample.

**Table 1:** Sex and age stratification of the sample

n = 1,187		Number of patients (%)
**Sex**	Male	657 (55.3%)
	Female	530 (44.7%)
**Age group**	4–5 years	167 (14.1%)
	6–10 years	442 (37.1%)
	11–20 years	427 (36%)
	21–30 years	98 (8.3%)
	31 years or more	53 (4.5%)

**Table 2:** Variable distribution among the groups, regarding parents and family history

		Bad quality of life	Good quality of life	*P* -value
**Fathers' educational background**	Illiterate	4.0%	1.1%	< 0.001
	Did not complete elementary school	28.9%	24.2%	
	Completed elementary school	38.4%	30%	
	Completed high school	24.9%	34.8%	
	Completed college or higher	3.8%	9.9%	
	Total	100%	100%	
**Mothers' educational background**	Illiterate	4.4%	1.4%	< 0.001
	Did not complete elementary school	32.1%	23.3%	
	Completed elementary school	36.3%	27.9%	
	Completed high school	24.1%	33.2%	
	Completed college or higher	3.1%	14.2%	
	Total	100%	100%	
**Maternal employment status**	Did not work	54.2%	36.8%	< 0.001
	Work	45.8%	60.2%	
	Total	100%	100%	
**Paternal employment status**	Did not work	4.1%	2.1%	0.6
	Work	95.9%	97.9%	
	Total	100%	100%	
**Family history of alcohol abuse**	Yes	90.9%	97.9%	< 0.001
	No	9.1%	2.1%	
	Total	100%	100%	
**Family history of psychiatric disorders**	Yes	21.7%	14.9%	0.02
	No	78.3%	85.1%	
	Total	100%	100%	

**Table 3:** Variable distribution among the groups, regarding patient's profile

		Bad quality of life	Good quality of life	*P-* value
**Age at 1st medical consult** **(median, in months)**		10	4.00	< 0.001
**Weight at birth (mean ± standard deviation, in grams)**		2,860.7 ± 541.4	2,832.7 ± 559.7	0.5
**Neonatal intercurrences**	Yes	42.9%	45.3%	0.76
	No	55.8%	55.3%	
**Apgar in the fifth minute**	< 7	1.2	3.2	0.51
	> 7	98.7	96.8	
**1st BERA**	Normal	79.2%	79.7%	0.84
	Altered	20.8%	20.3%	
	Total	100%	100%	
**BERA follow-up**	Normal	77.5%	77.5%	0.94
	Altered	22.5%	22.5%	
	Total	100%	100%	
**Audiometry**	Normal	91.9%	91.5%	0.97
	Altered	8.1%	8.5%	
	Total	100%	100%	
**Karyotype**	Regular trisomy	94.3%	92.4%	0.01
	Translocation	4.0%	4.4%	
	Mosaicism	1.6%	5.2%	
	Total	100%	100%	

Abbreviation: BERA, brainstem-evoked response audiometry.

**Table 4:** Comparison among factors in bad or good quality of life

	Autism		Epilepsy		ADHD	
	Absent	Present	Absent	Present	Absent	Present
**Bad quality of life**	87.1%	95.2%	94.1%	98.3%	98.4%	98.7%
**Good quality of life**	12.9%	4.8%	5.9%	1.7%	1.6%	1.0%
**Total**	100%	100%	100%	100%	100%	100%
***p*** **-value**	< 0.001		0.01		0.77	

Abbreviation: ADHD, attention deficit hyperactivity disorder.

Note: *Fisher's test.

The most common karyotype representation was simple or regular trisomy in 1,120 (94.4%) cases, followed by translocation in 31 (2.6%), and mosaicism in 28 (2.4%) patients. Simple trisomy was associated with another genetic abnormality in 7 (0.6%) patients, with Klinefelter syndrome being the most common overlapping chromosomal syndrome.

We observed that good quality of life was more frequently associated with female sex ( *p* = 0.01), higher parental educational level ( *p* < 0.001), mosaicism ( *p* = 0.001), adequate prenatal care ( *p* < 0.004), first medical consult in an earlier age ( *p* < 0.001), and mother employed ( *p* < 0.001). The paternal employment status did not present any statistically significant difference among the groups ( *p* = 0.6) ( Tables 2 and 3 ). In contrast, bad quality of life was mainly associated with a family history of alcohol abuse ( *p* < 0.001) and psychiatric conditions ( *p* = 0.02), and clinical comorbidities such as autism ( *p* < 0.001) and epilepsy ( *p* = 0.001) ( Table 4 ).

The variables weight at birth, neonatal intercurrences, APGAR in the 5 ^th^ minute, results in ancillary investigation (BERA and audiometry), karyotype (except mosaicism), cardiopathy, and attention deficit hyperactivity disorder did not present statistically significant differences among the groups ( Table 3 ).

## DISCUSSION

The current study gathered data on quality of life among Brazilian patients with DS comprehending a significant sample, reuniting self-reported information, views and perceptions from parents and caregivers, and data from their medical files. Most prior studies on the subject emphasized only the data reported by the parents. 7 In this cohort, a wide distribution between age groups was observed, but with a predominance of patients of school age and young adults. This demographic characterization might contribute to understanding how the patients reported predominantly bad quality of life, as most of the patients are either studying, working, or both, and the cognitive issues of the syndrome greatly impact academic and professional activities. A better quality of life was observed in females, similar to the described by Piper et al. 8 (1986), who reported better overall performance of children at 18 months in females. 8 The beneficial effect of parents presenting higher educational backgrounds and stable employment status was expected.

Contrary to general belief, children with working parents did not perform academically and cognitively worse. The reduced availability of time is often compensated by an improvement in the quality of time spent. 9 On the other hand, children of unemployed parents experience the detrimental effects of this status, as the absence of a steady income jeopardizes child support, impacts nutrition and educational quality, and might force children to abandon school and start working prematurely. 10 Parents with higher educational levels are more likely to have better jobs, which would allow an adequate income without the necessity of long working hours and demanding jobs that could impact the quality of time spent with their children. In addition, parents with better education are more prone to seek and obtain the educational reinforcement their children need, in addition to regular formal education, employing active learning methodologies. They also act as role models, indirectly stimulating their children to pursue higher education. 10 11 Gilmore et al. 12 observed that maternal educational strategies may have different consequences in children with and without DS. Children with DS whose mothers stimulate autonomy exhibited greater persistence in working independently in a challenging puzzle, while children of highly directive mothers had lower levels of persistence. For children with typical development, persistence was not related to maternal style. 12

Patients with DS greatly benefit from such educational strategies. One example is the language domain, in which a wide range of interventions can foster the acquisition of communication skills in children with DS, based on the diversity of free-time activities, regularly-scheduled activities, use of books and magazines, combined with adequate supervision of formal academic activities and a daily routine with defined schedules, emphasizing the importance of clear verbal communication. 12 Physical activity also plays a key role in the development of social skills and integration for patients with DS. Hardee et al. 13 reported in a systematic review the positive impact of exercise interventions on daily life activities and social participation for patients with DS. 13

In analyzing the factors that led to poor quality of life, the presence of clinical comorbidities, namely epilepsy and autism, was noteworthy. This finding was similar to the observed in the study by Haddad et al., but, in their cohort, quality of life was more impacted by bowel conditions and psychiatric disorders. 14 Fucà et al., in a cohort of 73 children with DS, also described a positive correlation between autistic symptoms and other behavioral conditions and low quality of life. 15

This study has certain limitations that are important to highlight. One major limitation of our report is the lack of standardization among the various research instruments used to assess quality of life, and the scarcity of studies in the context of DS further exacerbates this issue. Despite utilizing a validated quantitative tool such as the POS, the absence of clear cutoff points for quality of life in patients with DS poses a challenge. To address this, we employed a qualitative-quantitative approach and adapted the scale with specific parameters indicating good or poor quality of life. However, as expected in studies involving qualitative aspects, we heavily relied on patient and parental reports, which introduces the possibility of reporting bias. Although we gathered a significant sample, our data was derived from patients being followed up at a single center, thereby limiting the external validity of our study. Moreover, the high level of cultural heterogeneity in Brazil may influence the perception of quality of life and its associated factors.

In conclusion, clinical comorbidities such as autism and epilepsy carry a heavy burden among patients with DS, while factors related to family support, such as employment status and educational background of the parents, enhance quality of life. These factors should be considered when devising healthcare policies to improve cognitive, emotional, and social outcomes for patients with DS. More studies are necessary to capture the regional differences among Brazilian patients with DS.
